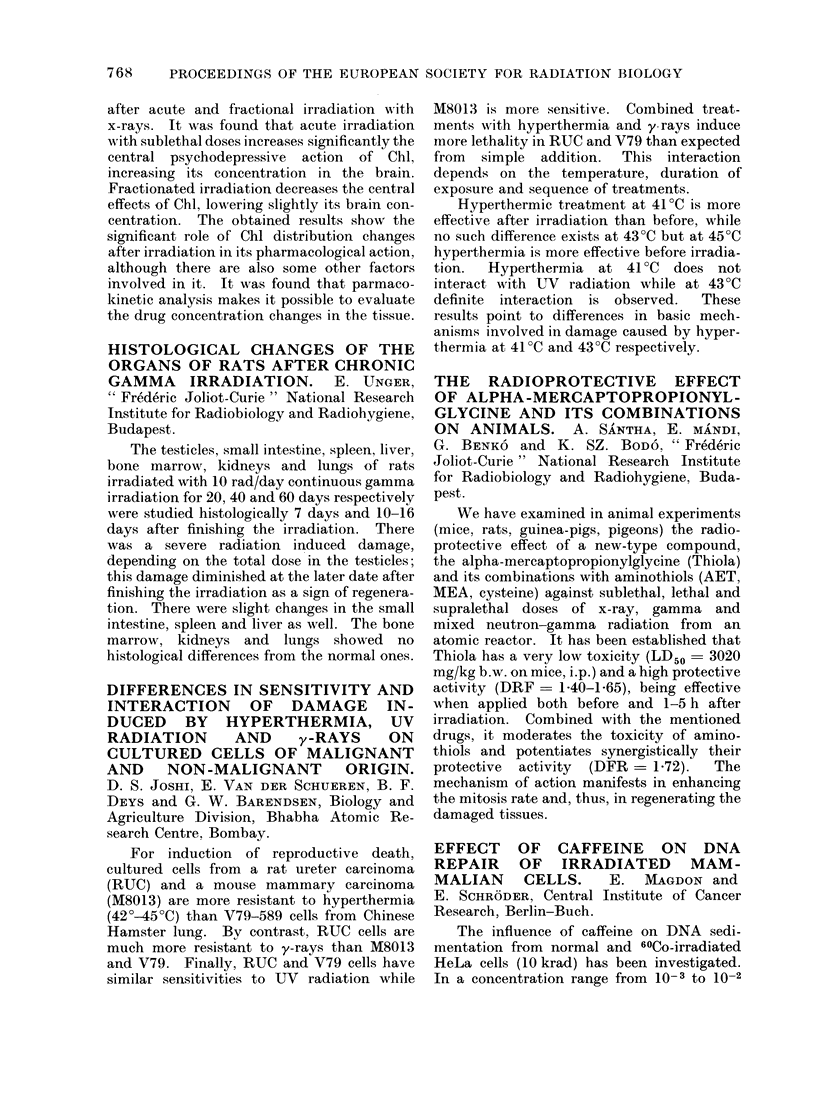# Proceedings: Differences in sensitivity and interaction of damage induced by hyperthermia, UV radiation and gamma-rays on cultured cells of malignant and non-malignant origin.

**DOI:** 10.1038/bjc.1975.346

**Published:** 1975-12

**Authors:** D. S. Joshi, E. Van der Schueren, B. F. Deys, G. W. Barendsen


**DIFFERENCES IN SENSITIVITY AND INTERACTION OF DAMAGE INDUCED BY HYPERTHERMIA, UV RADIATION AND $\gamma$-RAYS ON CULTURED CELLS OF MALIGNANT AND NON-MALIGNANT ORIGIN.** D. S. Joshi, E. Van der Schueren, B. F. Deys and G. W. Barendsen, Biology and Agriculture Division, Bhabha Atomic Research Centre, Bombay.

For induction of reproductive death, cultured cells from a rat ureter carcinoma (RUC) and a mouse mammary carcinoma (M8013) are more resistant to hyperthermia (42°–45°C) than V79–589 cells from Chinese Hamster lung. By contrast, RUC cells are much more resistant to $\gamma$-rays than M8013 and V79. Finally, RUC and V79 cells have similar sensitivities to UV radiation while M8013 is more sensitive. Combined treatments with hyperthermia and $\gamma$-rays induce more lethality in RUC and V79 than expected from simple addition. This interaction depends on the temperature, duration of exposure and sequence of treatments.

Hyperthermic treatment at 41°C is more effective after irradiation than before, while no such difference exists at 43°C but at 45°C hyperthermia is more effective before irradiation. Hyperthermia at 41°C does not interact with UV radiation while at 43°C definite interaction is observed. These results point to differences in basic mechanisms involved in damage caused by hyperthermia at 41°C and 43°C respectively.